# A Veterinary Twist on Pathogen Biology

**DOI:** 10.1371/journal.ppat.0030012

**Published:** 2007-02-23

**Authors:** Massimo Palmarini

**Affiliations:** The Scripps Research Institute, United States of America

The recent epidemics of bovine spongiform encephalopathy, foot and mouth disease, and avian influenza have focused the attention of the general public and scientific community on veterinary pathogens. Studies on naturally occurring infectious diseases of domestic animals, although perceived to be mostly relevant for animal health, have often unveiled new paradigms on the biology of infectious agents, inspired the identification of novel human pathogens, and occasionally launched entire new disciplines. This article aims to provide some examples that illustrate how veterinary diseases (focusing on viral diseases in particular) have provided novel comparative and biological platforms (Figure 1).

**Figure 1:**
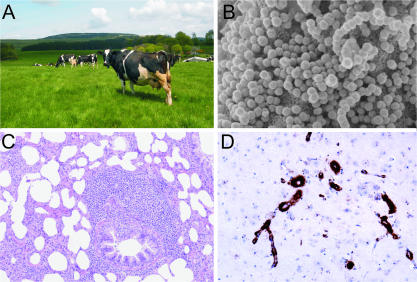
Novel Biological Platforms Unveiled in the Barnyard Studies on viruses of veterinary interest have revealed many fundamental aspects of pathogen biology. (A) Dairy cows grazing the Scottish hills. (B) Rous sarcoma virus particles budding from the surface of chicken fibroblasts as imaged by scanning electron microscopy. (C) Histology from a lung section of a sheep affected by maedi-visna virus showing chronic interstitial pneumonia. (D) Immunohistochemistry showing disease-associated prion protein (PrP) vascular amyloid in the cerebellar cortex of a sheep affected by scrapie.

The discipline of animal virology itself began with the discovery of the causative agents of major infectious diseases of domestic animals: foot and mouth disease initially [1], followed by the recognition of “filterable agents” in rabbit myxomatosis, African horse sickness, avian influenza (fowl plague), and avian leukosis [2,3]. Soon after, Peyton Rous [4,5] demonstrated the viral etiology of avian sarcoma, a discovery met with great skepticism at the time, as it was not believed that cancer could be infectious. Curiously, modern cancer genetics was essentially initiated with studies on the very same virus (Rous sarcoma virus) discovered by Rous (Figure 1B). The genome of Rous sarcoma virus, in addition to the canonical retroviral structural and enzymatic genes, was found to contain a cell-derived gene, *v-src*, which is both the first discovered oncogene and the first tyrosine kinase [6–9]! Over the years, avian retroviruses have been instrumental to the discovery of many other oncogenes such as *myc, jun, myb, rel, erbA,* and others that are now considered “usual suspects” in cancer initiation and progression [10].

The above examples are described in most biology textbooks along with the pioneer studies of Jenner, Koch, and Pasteur on cowpox, anthrax, and rabies. However, there are instances where studies on viral diseases of domestic animals had a profound impact on biology and medicine that is not often appreciated outside veterinary science. Rinderpest (also known as “cattle plague”), for example, has a rich and long history of scientific landmarks [11]. The consequences of rinderpest for agriculture were so serious that in the 18th century, Pope Clement XI instructed his physician, Giovanni Maria Lancisi, to devise measures to control the disease. Lancisi declared that the disease “was caused by exceedingly fine and pernicious particles that pass from one body to another”. He made the suggestion to “destroy all ill and suspect animals . . . rather than allowing the contagion to increase . . .” and to bury the whole animals in lime [11]. These principles constituted a milestone in controlling the spread of contagious diseases by restrictions on animal movements, quarantine, and removal of affected animals.

In 1762, the first veterinary school in the world opened in Lyon (France) in order to teach the principles put forward by Lancisi, who is appropriately considered the first modern hygienist. Three hundred years later, we have faced and controlled the epidemic of foot and mouth disease in the United Kingdom by using essentially the same measures devised by Lancisi to control rinderpest.

Rinderpest is associated with other scientific milestones. A self-taught Dutch farmer, Geert Reinders, during a rinderpest epidemic in 1768, was the first to grasp the concept of “maternal immunity” and to notice that cattle which had experienced the natural illness (or mild symptoms) were protected from subsequent infection and disease [3,12]. This observation was published well before Jenner's landmark studies on cowpox [3,12]. Curiously, the clinical thermometer was developed in order to diagnose fever in cattle suspected to be affected by rinderpest as an additional tool to control the disease [12].

Studies during the 1940s and 1950s by Björn Sigurdsson on a small group of diseases of sheep bred in Iceland [13] constitute a true biological “treasure”. Maedi-visna, ovine pulmonary adenocarcinoma (OPA), and paratuberculosis were introduced into Iceland after the import of a small number of rams from Germany in 1933. These diseases spread in the Icelandic flocks despite the fact that imported rams were regularly kept in quarantine on the island before putting them in contact with the local sheep population. Maedi-visna, OPA, and paratuberculosis had been described before [14,15], but the unique epidemiological and geographical circumstances surrounding the Icelandic epidemics stimulated Sigurdsson to develop the concept of “slow diseases” of sheep, in which an infectious agent could induce disease in its host months or even years after initial infection. Sigurdsson also recognized scrapie, present since 1878 in Iceland, as one of the slow diseases of sheep [13].

Over the intervening years, these relatively obscure diseases of small ruminants had several claims to fame in the biomedical arena. Maedi-visna, for example, a disease characterized mainly by pneumonia and encephalitis, was subsequently discovered to be caused by a retrovirus [16]. This retrovirus, maedi-visna virus (Figure 1C), was considered the prototype of the *Lentivirus* genus within the Retroviridae family. The name “lentivirus” derives from the Latin for “slow” and refers to the term coined by Sigurdsson. Another lentivirus, HIV, would emerge afterwards as the cause of AIDS and soon became the most studied virus in human history. Early studies on HIV-1, now the new lentivirus prototype, in the 1980s took some advantage on data accumulated on maedi-visna virus and on another animal lentivirus, equine infectious anemia virus [17–20].

Scrapie, another of the slow diseases of sheep, was the first disease described [15] within the group of the transmissible spongiform encephalopathies (TSEs) (Figure 1D). The apparent lack of nucleic acids in the infectious material associated with scrapie and other TSEs has represented a wonderful biological puzzle, culminating in the award of the Nobel prize to Stanley Prusiner for the prion theory [21]. TSEs have also been diagnosed in humans and include kuru, Gerstmann-Sträussler-Scheinker syndrome, fatal familial insomnia, Creutzfeldt-Jakob disease, and the new variant Creutzfeldt-Jakob disease. Kuru, the first recognized human TSE, was originally described by Carleton Gajdusek and Vincent Zigas in members of the Fore tribe in the Papua New Guinea at the end of the 1950s. Interestingly, the first hint that kuru and scrapie might be similar diseases was made serendipitously by a veterinary pathologist, William Hadlow, who was attending an exhibit on kuru at the Wellcome Medical Museum in London [22]. The vacuolated neuronal cell bodies were unusual in human pathology but well characterized in sheep with scrapie. Seven years after this connection was made, Gajdusek and colleagues described the experimental transmission of kuru in chimpanzees inoculated with brain tissues from persons who had died of the disease [23].

Of course, when the bovine spongiform encephalopathy epidemic spread across the UK in the 1990s [24] and public health concerns started to rise, the early studies on scrapie provided a critical intellectual framework (although possibly misleading with respect to potential human transmission) to understand the biology of bovine spongiform encephalopathy and the zoonotic variant Creutzfeldt-Jakob disease [25,26].

The other slow disease of sheep, OPA, is probably best known to the general public for being the cause of the demise of Dolly, the first mammal cloned by nuclear transfer. However, the causative agent of OPA, a retrovirus known as Jaagsiekte sheep retrovirus (JSRV), has been found to be the only oncogenic retrovirus (and the only oncogenic virus in general) to possess a structural protein (the viral envelope) functioning as a dominant oncogene in vitro and in vivo [27–29]. In addition, the JSRV/OPA model sparked fundamental studies on the biological roles of endogenous retroviruses in mammalian evolution. Endogenous retroviruses are ancient remains of retrovirus infections stably integrated in the genome of every animal species. Studies on sheep endogenous retroviruses related to JSRV demonstrated in experiments in vivo that endogenous retroviruses can play a fundamental role in host placentation and conceptus development [30].

Many viruses of domestic animals have also inspired, directly or indirectly, discoveries of related human viruses. Feline leukemia virus [31,32] was taken as an example that retroviruses horizontally transmitted could cause tumours in outbred animal species; this fuelled the chase for human retroviruses, eventually leading to the discovery of human T lymphotropic virus [33,34].

An interesting example of viruses identified in animals before being found in humans is provided by rotaviruses, the main cause of diarrhea in infants and children throughout the world and in the developing countries in particular [35–37]. The causal association between rotaviruses and diarrhea was made in calves well before it was realized for humans [37,38]. Mebus and colleagues demonstrated that diarrhea in calves was caused by rotaviruses [39,40] and established a cell culture system to propagate them [41]. In addition, studies in both calves and lambs were key to realizing the importance of local immunity and colostrum-derived antibodies in the resistance to rotavirus infection [42,43]. All of these studies have had a profound impact in understanding and preventing human rotavirus infection.

Studies on animal papillomaviruses of rabbits, cattle, and dogs have provided the intellectual background for the identification of human papillomaviruses as etiological agents of the overwhelming majority of cervical cancers and some cutaneous cancers [44–48]. The success of the recently highly publicized vaccine against human papillomavirus is based on the proof of principle gained in vaccine development against animal papillomaviruses [49].

Animal viruses are also important causes of zoonosis. The recent zoonotic episodes of Nipah virus, Hendra virus [50], and, more importantly, avian influenza [51–53], are a growing list of examples on infectious agents that can pass from animals to humans without previous “warning”, a particularly worrisome scenario given that 75% of human infections are estimated to be zoonotic in origin [54].

This article has no pretensions to be exhaustive but aims to show how research on pathogens of veterinary interest needs to be fostered, not only for its direct relevance to animal health, but for its significance to comparative medicine and public health. Veterinary scientists play important roles in the study of infectious diseases and can operate at the interface between basic, applied, and clinical research since they are equipped with a unique combination of expertise in biology, ecology, and husbandry [55]. The understanding of complex emerging diseases needs collaborative efforts between experts in public health, the environment, and animal health, where veterinary scientists can provide a critical contribution [56].

Unfortunately, in the last two decades there has been a steady decline worldwide in the number of veterinary graduates undertaking a career in research, which is a cause of great concern. Veterinary schools and veterinary research institutes should create an environment where hypothesis-driven research is encouraged and maintained, and its importance continuously highlighted to funding agencies, policy makers, veterinary students, and the scientific community as a whole. 

## Abbreviations

OPA: ovine pulmonary adenocarcinoma
TSE: transmissible spongiform encephalopathy

## References

[ppat-0030012-b001] Loeffler F, Frosch P (1897). Summarischer bericht über die ergebnisse der untersuchungen der Kommission zur Erforschung der maul und klauenseuche bei dem Institut für Infektionskrankheiten. Dt Med Wschr.

[ppat-0030012-b002] Ellerman V, Bang O (1908). Experimentelle leukamie bei huhnern. Fizentralblatt bakteriologie. Zentralblatt der Bakteriologie.

[ppat-0030012-b003] Horzinek MC (1997). The birth of virology. Antonie Van Leeuwenhoek.

[ppat-0030012-b004] Rous P (1911). Transmission of a malignant new growth by means of a cell-free filtrate. JAMA.

[ppat-0030012-b005] Rous P (1911). A sarcoma of the fowl transmissible by an agent separable from the tumour cells. J Exp Med.

[ppat-0030012-b006] Duesberg PH, Vogt PK (1970). Differences between the ribonucleic acids of transforming and nontransforming avian tumor viruses. Proc Natl Acad Sci U S A.

[ppat-0030012-b007] Stehelin D, Guntaka RV, Varmus HE, Bishop JM (1976). Purification of DNA complementary to nucleotide sequences required for neoplastic transformation of fibroblasts by avian sarcoma viruses. J Mol Biol.

[ppat-0030012-b008] Stehelin D, Varmus HE, Bishop JM, Vogt PK (1976). DNA related to the transforming gene(s) of avian sarcoma viruses is present in normal avian DNA. Nature.

[ppat-0030012-b009] Czernilofsky AP, Levinson AD, Varmus HE, Bishop JM, Tischer E (1980). Nucleotide sequence of an avian sarcoma virus oncogene (src) and proposed amino acid sequence for gene product. Nature.

[ppat-0030012-b010] Rosenberg N, Jolicoeur P, Coffin JM, Hughes S, Varmus HE (1997). Retroviral pathogenesis. Retroviruses.

[ppat-0030012-b011] Barrett T, Pastoret PP, Taylor W (2005). Rinderpest and peste des petits ruminants: Virus plagues of large and small ruminants.

[ppat-0030012-b012] Spinage C (2003). Cattle plague: A history.

[ppat-0030012-b013] Sigurdsson B (1954). Rida, a chronic encephalitis of sheep: With general remarks on infections which develop slowly and some of their special characteristics. Br Vet J.

[ppat-0030012-b014] McFadyean J (1894). Verminous pneumonia in the sheep. J Comp Pathol Ther.

[ppat-0030012-b015] Stockman S (1913). Scrapie: An obscure disease of sheep. J Comp Pathol.

[ppat-0030012-b016] Lin FH, Thormar H (1970). Ribonucleic acid-dependent deoxyribonucleic acid polymerase in visna virus. J Virol.

[ppat-0030012-b017] Clements JE, Gdovin SL, Montelaro RC, Narayan O (1988). Antigenic variation in lentiviral diseases. Annu Rev Immunol.

[ppat-0030012-b018] Narayan O, Zink MC, Huso D, Sheffer D, Crane S (1988). Lentiviruses of animals are biological models of the human immunodeficiency viruses. Microb Pathog.

[ppat-0030012-b019] Haase AT (1986). Pathogenesis of lentivirus infections. Nature.

[ppat-0030012-b020] Thormar H (2005). Maedi-visna virus and its relationship to human immunodeficiency virus. AIDS Rev.

[ppat-0030012-b021] Prusiner SB (1991). Molecular biology of prion diseases. Science.

[ppat-0030012-b022] Hadlow WJ (1999). Reflections on the transmissible spongiform encephalopathies. Vet Pathol.

[ppat-0030012-b023] Liberski PP, Gajdusek DC (1997). Kuru: Forty years later, a historical note. Brain Pathol.

[ppat-0030012-b024] Hope J, Reekie LJ, Hunter N, Multhaup G, Beyreuther K (1988). Fibrils from brains of cows with new cattle disease contain scrapie-associated protein. Nature.

[ppat-0030012-b025] Wilesmith JW (1988). Bovine spongiform encephalopathy. Vet Rec.

[ppat-0030012-b026] Wilesmith JW, Wells GA, Cranwell MP, Ryan JB (1988). Bovine spongiform encephalopathy: Epidemiological studies. Vet Rec.

[ppat-0030012-b027] Maeda N, Palmarini M, Murgia C, Fan H (2001). Direct transformation of rodent fibroblasts by Jaagsiekte sheep retrovirus DNA. Proc Natl Acad Sci U S A.

[ppat-0030012-b028] Wootton SK, Halbert CL, Miller AD (2005). Sheep retrovirus structural protein induces lung tumours. Nature.

[ppat-0030012-b029] Caporale M, Cousens C, Centorame P, Pinoni C, De las Heras M (2006). Expression of the Jaagsiekte sheep retrovirus envelope glycoproteins is sufficient to induce lung tumor in sheep. J Virol.

[ppat-0030012-b030] Dunlap KA, Palmarini M, Varela M, Burghardt RC, Hayashy K (2006). Endogenous retroviruses regulate peri-implantation conceptus growth and differentiation. Proc Natl Acad Sci U S A.

[ppat-0030012-b031] Jarrett WF, Crawford EM, Martin WB, Davie F (1964). A virus-like particle associated with leukemia (lymphosarcoma). Nature.

[ppat-0030012-b032] Jarrett WF, Martin WB, Crighton GW, Dalton RG, Stewart MF (1964). Transmission experiments with leukemia (lymphosarcoma). Nature.

[ppat-0030012-b033] Gallo RC, Kalyanaraman VS, Sarngadharan MG, Sliski A, Vonderheid EC (1983). Association of the human type C retrovirus with a subset of adult T-cell cancers. Cancer Res.

[ppat-0030012-b034] Gallo RC (2005). History of the discoveries of the first human retroviruses: HTLV-1 and HTLV-2. Oncogene.

[ppat-0030012-b035] Parashar UD, Bresee JS, Gentsch JR, Glass RI (1998). Rotavirus. Emerg Infect Dis.

[ppat-0030012-b036] Parashar UD, Kilgore PE, Holman RC, Clarke MJ, Bresee JS (1998). Diarrheal mortality in US infants. Influence of birth weight on risk factors for death. Arch Pediatr Adolesc Med.

[ppat-0030012-b037] Kapikian AZ, Hoshino Y, Chanok RM, Knipe DM, Howley PM (2001). Rotaviruses. Fields virology. 4th edition.

[ppat-0030012-b038] Bishop RF, Davidson GP, Holmes IH, Ruck BJ (1973). Virus particles in epithelial cells of duodenal mucosa from children with acute non-bacterial gastroenteritis. Lancet.

[ppat-0030012-b039] Mebus CA, Underdahl NR, Rhodes MB, Twiehaus MJ (1969). Further studies on neonatal calf diarrhea virus. Proc Annu Meet U S Anim Health Assoc.

[ppat-0030012-b040] Mebus CA, Underdahl NR, Rhodes M, Twiehaus M (1969). Calf diarrhea (scours): Reproduced with a virus from field outbreak. Univ Nebraska Res Bull.

[ppat-0030012-b041] Mebus CA, Kono M, Underdahl NR, Twiehaus MJ (1971). Cell culture propagation of neonatal calf diarrhea (scours) virus. Can Vet J.

[ppat-0030012-b042] Snodgrass DR, Wells PW (1976). Rotavirus infection in lambs: Studies on passive protection. Arch Virol.

[ppat-0030012-b043] Woode GN, Jones J, Bridger J (1975). Levels of colostral antibodies against neonatal calf diaahoea virus. Vet Rec.

[ppat-0030012-b044] Nicholls PK, Stanley MA (1999). Canine papillomavirus—A centenary review. J Comp Pathol.

[ppat-0030012-b045] Rous P, Kidd JG (1938). The carcinogenic effect of a papillomavirus on the tarred skin of rabbits. J Exp Med.

[ppat-0030012-b046] Jarrett WF, Murphy J, O'Neil BW, Laird HM (1978). Virus-induced papillomas of the alimentary tract of cattle. Int J Cancer.

[ppat-0030012-b047] Campo MS (2002). Animal models of papillomavirus pathogenesis. Virus Res.

[ppat-0030012-b048] Gissmann L, Boshart M, Durst M, Ikenberg H, Wagner D (1984). Presence of human papillomavirus in genital tumors. J Invest Dermatol.

[ppat-0030012-b049] Giles M, Garland SM, Campo MS (2006). HPV vaccines. Papillomavirus biology: From natural history to vaccine and beyond.

[ppat-0030012-b050] Eaton BT, Broder CC, Middleton D, Wang LF (2006). Hendra and Nipah viruses: Different and dangerous. Nat Rev Microbiol.

[ppat-0030012-b051] Fauci AS (2006). Emerging and re-emerging infectious diseases: Influenza as a prototype of the host-pathogen balancing act. Cell.

[ppat-0030012-b052] Webster RG, Peiris M, Chen H, Guan Y (2006). H5N1 outbreaks and enzootic influenza. Emerg Infect Dis.

[ppat-0030012-b053] Capua I, Alexander DJ (2004). Human health implications of avian influenza viruses and paramyxoviruses. Eur J Clin Microbiol Infect Dis.

[ppat-0030012-b054] Taylor LH, Latham SM, Woolhouse ME (2001). Risk factors for human disease emergence. Philos Trans R Soc Lond B Biol Sci.

[ppat-0030012-b055] Brown C (2006). Avian influenza: Virchow's reminder. Am J Pathol.

[ppat-0030012-b056] Capua I, Brown I, Johnson M, Senne D, Swayne D (2006). Veterinary virologists share avian flu data. Science.

